# Assessment of Level of Depression and Associated Factors among COVID-19-Recovered Patients: a Cross-Sectional Study

**DOI:** 10.1128/spectrum.04651-22

**Published:** 2023-02-08

**Authors:** Khadeza Khatun, Nasreen Farhana

**Affiliations:** a Department of Hospital Management, National Institute of Preventive and Social Medicine (NIPSOM), Dhaka, Bangladesh; b Department of Microbiology and Mycology, National Institute of Preventive and Social Medicine (NIPSOM), Dhaka, Bangladesh; Oklahoma State University

**Keywords:** Bangladesh, COVID-19-recovered patients, depression, NIPSOM, PHQ-9 scale

## Abstract

The coronavirus disease 2019 (COVID-19) pandemic has psychological consequences such as increased risk of depression, anxiety, and stress problems, exacerbating human health disparities. This study aimed to analyze depression and its causes in COVID-19-recovered patients in Bangladesh. A cross-sectional study was conducted on COVID-19-recovered patients who attended follow-up visits after 14 days to 3 months at Dhaka Medical College Hospital (DMCH) and Dhaka North City Corporation Hospital (DNCCH), Dhaka, Bangladesh, from 1 January to 31 December 2021. Respondents were face-to-face interviewed with a semistructured questionnaire after written agreement. The Patient Health Questionnaire (PHQ-9) was used to assess respondents’ depression, and data were analyzed using SPSS version 23, with a *P* value of <0.05 indicating statistical significance. A total of 325 COVID-19-recovered patients aged from 15 to 65 years (mean, 44.34 ± 13.87 years) were included in this study, the highest proportion (23.1%) of them were aged 46 to 55 years, and the majority (61.5%) of them were male. There were 69.5% of respondents who had no signs of depression while 31% of them did have signs, with 26.7% being mildly depressed, 2.5% being extremely depressed, and 1.2% being severely depressed. Diabetes mellitus, hospitalization duration, social distancing, social media posts on COVID-19, loss of employment, family damage, and fear of reinfection were significantly associated with depression level of respondents. This study gives us a glimpse into the psychological health of COVID-19-recovered patients, and its findings highlight the imperative of alleviating their psychological anguish in Bangladesh.

**IMPORTANCE** The COVID-19 pandemic had a significant psychological impact on healthy populations, with increased depression, perceived stress, posttraumatic stress disorder, and insomnia reported. The COVID-19 pandemic affects people’s mental health by instilling fear of infection and depression. In the post-COVID-19 syndrome, depressive symptoms and clinically significant depression may have serious consequences for quality-of-life outcomes. To combat the spread of COVID-19, the Bangladesh government has implemented a number of measures, including lockdown, social distancing, self-isolation, and quarantine. Given the negative consequences, it is critical to investigate potential factors and mechanisms that may shed light on mental health improvement. The purpose of the study is to determine the level of depression in patients 3 months after recovering from acute COVID-19. The study’s findings highlight the need for COVID-19-infected people in Bangladesh to receive health education and interventions.

## INTRODUCTION

Since the dawn of 2020, the world has faced a pandemic caused by SARS-CoV-2, the highly contagious COVID-19 (1). Bangladesh has been suffering from this highly transmissible disease since March 2020 (2). The rapid spread of the disease in a wide variety of people makes them subject to varied degrees of panic, which makes it difficult to treat and rehabilitate them (3). According to earlier studies, viral respiratory infections are linked to chronic and acute psychological effects among the survivors including posttraumatic stress, insomnia, depression, anxiety, and even suicidality. After discharge, many of these patients’ mental disturbances persisted and continued for a long time (4, 5).

The COVID-19 pandemic has a significant psychological impact on healthy populations, with an increase in depression, perceived stress, posttraumatic stress, and insomnia being reported (6). The COVID-19 pandemic situation is fast-changing with worldwide case counts of crisis, suicide, domestic violence, mental disorder, anxiety, and depressive disorders (7). To combat the spread of COVID-19, the Bangladesh government has implemented a number of measures, including lockdown, social distancing, self-isolation, and quarantine (8). Besides, recent findings have demonstrated prevailing acute psychiatric symptoms among patients while being treated in isolation, lacking interaction with friends, family, or loved ones, being admitted to intensive care units (ICUs), and requiring mechanical ventilation (9). Extreme fear, when accompanied with social and economic implications (e.g., job loss, reduced earnings, relationship problems), has the potential to encourage individuals to engage in irrational thinking, which can lead to psychological discomfort (10).

In prior lethal viral epidemics, fear of death, loneliness, boredom, anxiety, sadness, social isolation, contagion, and (in severe situations) thoughts of suicide might result in long-term psychological repercussions among the general population (11). In the present COVID-19 pandemic, depression and anxiety problems are more activated. A recent systematic review reported that the pooled prevalence of depression and anxiety among COVID-19 patients was 45% and 47%, respectively (12).

Patients with COVID-19 have different degree of psychological pain such as anxiety and depression, which may worsen their prognosis by negatively affecting the patients’ immunity. As a result, clinically significant depression could have serious consequences for quality of life (13). Despite its increasing significance, present estimates on the prevalence of psychological distress, such as anxiety and depressive symptoms, among COVID-19 patients are uncertain (12).

In the aftermath of a natural disaster, a high rate of mental health suffering (i.e., a prevalence rate of 65% depression, much higher than rates reported elsewhere) was previously reported and appears to reflect the vulnerability of the Bangladeshi population to mental health suffering in a pandemic situation such as COVID-19 (14). During pandemics, public health officials and the media are more concerned with the biological and physical consequences of the outbreak than with mental health difficulties. With an increasing number of reports indicating that the COVID-19 outbreak is causing an increase in mental health burden, there have been greater calls for steps to improve mental health support for the public (15).

To date, scant findings on COVID-19 have been reported regarding the prevalence and associated factors of depression among COVID-19=recovered patients in Bangladesh. Therefore, this study aimed to comprehensively assess the level of depression and explore the factors associated with depressive symptoms 3 months after recovery from COVID-19 infection.

## RESULTS

### Sociodemographic characteristics of respondents.

This study comprised a total of 325 respondents, whose ages ranged from 15 to 65 years. The respondents’ mean age was 44.34 years, with standard deviation (SD) of ±13.87 years, and the highest proportion (23.1%) were between 46 and 55 years old. The majority of respondents (61.5%) were men, 75.7% were married, 92% were Muslims, 21.2% were graduates, 31.4% were business owners, 81.5% belonged to nuclear families, 61.5% resided in urban areas, 89.2% of them had only one family member who was financially contributing, 37% of respondents did not complete vaccination against COVID-19, and hypertension was found to be the most prevalent chronic disease (Table 1).

**TABLE 1:** Sociodemographic characteristics of the respondents

Characteristic	Frequency (*f*)	%
Age (yr)		
15–25	37	11.4
26–35	72	22.2
36–45	65	20.0
46–55	75	23.1
56–65	65	20.0
66–75	11	3.4
Mean (±SD)	44.34 ± 13.87	
Sex		
Male	200	61.5
Female	125	38.5
Marital status		
Married	246	75.7
Single	45	13.8
Widowed	31	9.5
Separated	2	0.6
Divorced	1	0.3
Religion		
Islam	299	92.0
Hinduism	25	7.7
Christianity	1	0.3
Educational qualification		
Illiterate	64	19.7
Primary	65	20.0
Secondary	55	16.9
Higher secondary	49	15.1
Graduation	69	21.2
Postgraduation	23	7.1
Occupation		
Government employee	30	9.2
Private employee	51	15.7
Businessperson	102	31.4
Homemaker	81	24.9
Student	34	10.5
Type of family		
Nuclear family	265	81.5
Extended family	60	18.5
Residency		
Urban	200	61.5
Rural	125	38.5
Income-earning family member(s)		
Self or 1	290	89.2
More than 1	35	10.8
History of chronic disease		
Diabetes mellitus	91	28.0
Cardiac disease	20	6.2
Asthma or COPD^*a*^	66	20.3
Kidney disease	13	4.0
Hypertension	107	32.9
History of vaccination against COVID-19		
Two doses completed	205	63.1
Not completed	120	36.9

a COPD, chronic obstructive pulmonary disease.

### Distribution of conditions of COVID-19-recovered patients.

One-fourth of 325 respondents (25.2%) had personal life interruptions and 78.2% had physical symptoms after COVID-19, primarily weakness (57%) and cough (31%). Of them, 4.3% were taking antidepressants, 1.2% had a family history of depression, 12% were hospitalized for more than 21 days, 10.8% needed ICU, and 12.3% were distressed due to social distancing. Also, 10.2% were affected by social media posts about COVID-19, 13% felt harmful to the family, 2.8% feared losing their jobs, 6.2% feared reinfection with COVID-19, and 29% had relatives or acquaintances who died from COVID-19 (Table 2).

**TABLE 2:** Distribution of conditions of COVID-19-recovered patients (*n* = 325)

Variable	Frequency	%
Interruption of personal life	82	25.2
Physical symptoms after recovery	254	78.2
Physical symptoms		
Dyspnea	63	19.4
Weakness	184	56.6
Cough	101	31.1
Fatigue	85	26.2
Muscle pain	30	9.2
Palpitation	45	13.8
Taking antidepressant drug	14	4.3
Family history of depression	4	1.2
Duration of hospitalization (days)		
1–7	183	56.3
7–14	61	18.8
14–21	33	10.2
More than 21 days	39	12.0
Needing ICU	35	10.8
Mental distress due to social distancing	40	12.3
Being affected by posts on social media about COVID-19	33	10.2
Fear of being harmful to one’s family	42	12.9
Fear of losing job	9	2.8
Fear of reinfection with COVID-19	20	6.2
Death of relative(s) or acquaintance(s) from COVID-19	93	28.6

### Prevalence of depression.

**FIG 1:**
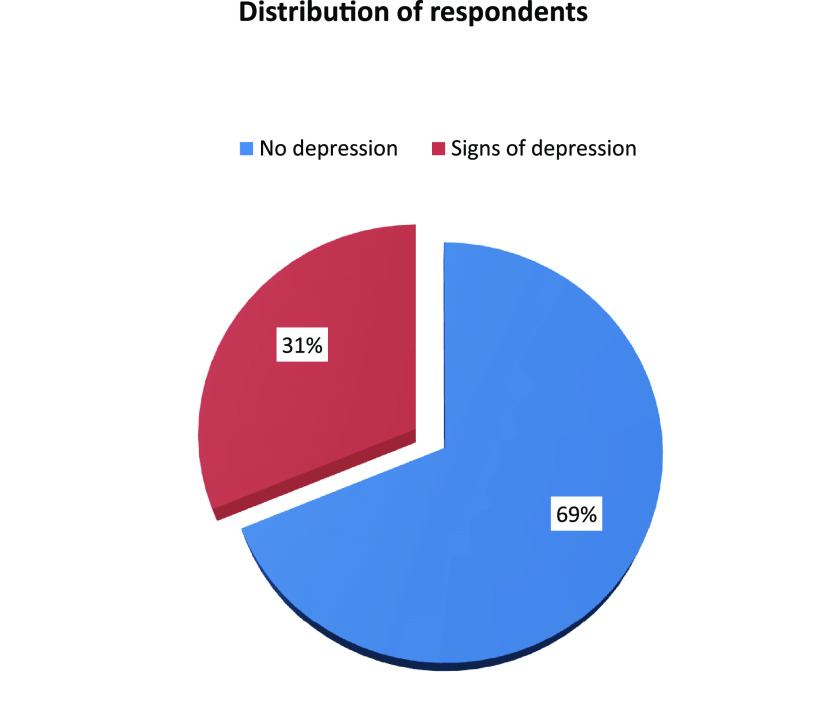
Distribution of respondents according to signs of depression (*n* = 325).

The prevalence of depression was assessed by a PHQ-9 scale comprising 9 items (Table 3) showing that about one-third of respondents (31%) had symptoms of depression (Fig. 1), whereas 87.8% of them were mildly depressed, 8.1% were moderately depressed, and 4.0% were severely depressed (Table 4).

**TABLE 3:** Depression=related questions according to patient health questionnaire PHQ-9 (9 items) (*n* = 325)

Attribute	No. (%) of respondents with frequency:			
	Not at all	Several days	More than half of days	Nearly every day
Little interest or pleasure in doing things	244 (75.1)	19 (5.8)	27 (8.3)	35 (10.8)
Feeling down or hopeless	226 (69.5)	51 (15.7)	19 (5.8)	29 (8.9)
Trouble falling or staying asleep or sleeping too much	139 (42.8)	74 (22.8)	8 (2.5)	104 (32)
Feeling tired or having little energy	103 (31.7)	81 (24.9)	6 (1.8)	135 (41.5)
Poor appetite or overeating	149 (45.8)	48 (14.8)	6 (1.8)	122 (37.5)
Feeling bad about oneself	292 (89.8)	11 (3.4)	18 (5.5)	4 (1.2)
Trouble concentrating	283 (87.1)	25 (7.7)	3 (0.9)	14 (4.3)
Moving or speaking slowly	271 (83.4)	44 (13.5)	6 (1.8)	4 (1.2)
Thoughts that one would be better off dead	307 (94.5)	6 (1.8)	3 (0.9)	9 (2.8)

**TABLE 4:** Distribution of respondents according to level of depression (*n* = 99)

Level of depression	Frequency (*f*)	%
Mild	87	87.9
Moderate	8	8.1
Severe	4	4.0

### Association of prevalence of depression with variables of respondent.

The variables of educational qualification, diabetes mellitus history, duration of hospitalization, distress due to social distancing, being affected by posts on social media about COVID-19, fear of job loss, feeling harmful to family members, fear of reinfection, taking an antidepressant drug, and interruption of personal life were significantly associated with the prevalence of depression (*P* < 0.05) (Table 5).

**TABLE 5:** Association of level of depression with variables of respondent (*n* = 325)

Association	Test value	*P* value
Sex of respondent	3.081	0.379
Educational qualification	36.701	0.001
History of diabetes mellitus	13.005	0.003
Duration of hospitalization	19.520	0.007
Mental distress due to social distancing	28.881	0.001
Being affected by posts on social media	32.667	0.001
Fear of losing job	10.780	0.013
Fear of being harmful to family members	29.990	0.001
Fear of further contamination	13.715	0.003
Receiving antidepressant drug	11.550	0.006
Interruption of personal life	48.963	0.001

## DISCUSSION

This study found that among 325 respondents, 31% had depression symptoms, with 26.8% exhibiting mild symptoms, 2.5% exhibiting moderate symptoms, and 1.2% exhibiting severe symptoms. Several studies were undertaken in Bangladesh following the commencement of COVID-19 to assess mental health, including depression. A cross-sectional study was conducted at the initial stage after the COVID-19 outbreak (from 1 June to 10 June 2020) among 1,146 Bangladeshi participants by Zubayer et al. (16), in which 47.2% of participants had depression, with 16.2% being mildly depressed, 20.4% moderately depressed, and 6.5% severely depressed, rates which were higher than those in this study. Another study, conducted by Das et al. (17), among 672 Bangladeshi people aged between 15 and 65 years from 15 April to 10 May 2020 reported a prevalence of depression of 38% with 24% being mildly depressed, 11% moderately depressed, and 3% severely depressed. Another study conducted among Bangladeshi students during the COVID-19 pandemic reported the prevalence rates of depression to be 46.9% (18). The increased prevalence of depression could be attributed to the virus’s ongoing transmission, the increasing number of new cases, the death of a loved one, and the fear of infection during the early stages of the COVID-19 outbreak, when individuals were challenged by mandatory quarantine, unexpected unemployment, and uncertainty associated with the outbreak (16, 18).

According to certain research, depressive symptoms were recorded at lower prevalence rates than in the present study, such as 16.5% of the Chinese population (19), 11.4% of Japanese people (20), 17.3% of Italians (3), 3.7% of Portuguese people (21), and 26% of Iranian people (22). Prevalence rates varied between studies, which could be explained by differences in government preparedness, the availability of medical supplies and facilities, the effective communication of information about COVID-19, or international/cultural variances affecting the psychological health of the general public (18).

According to the results of this study, the highest proportion (23.1%) of the respondents were between the ages of 46 and 55 years. This is because the risk of COVID-19-related illness and mortality increases with age (8). In some studies, individuals under the age of 45 had more adverse psychological symptoms during the pandemic (23, 24). This is consistent with our findings that about 54% of the participants were below 45 years of age. This finding could be explained in part by their role as family caregivers, who provide financial and emotional support to children and the elderly. Among the respondents, 61.5% were male, although females have been linked to mental health issues, but there were no significant sex differences (*P* = 0.379) in regard to depression in the current investigation (18, 25). Because of the COVID-19 condition, sex disparities in this study may be negated.

COVID-19 is highly contagious and can be passed from person to person (26). Therefore, individuals fear either having COVID-19 themselves or becoming asymptomatic carriers who spread the disease unknowingly to family and friends, contributing to psychiatric symptoms (21, 26). In line with previous studies, the present study also showed that fear of infection is significantly associated with depression (*P* = 0.001) (23, 27). That the death of a loved one leads to psychological problems, such as depression, is supported by the findings of the present study (26).

The present study showed that daily exposure to COVID-19-related news was significantly associated with overall mental health problems. Previous studies showed that people who were exposed to COVID-19-related news were more likely to develop psychiatric symptoms (23, 28). Moreover, symptoms of depression and anxiety among COVID-19 inpatients could increase due to uncertainty about the prognosis of the disease and the experience of adverse outcomes (16). Furthermore, side effects of COVID-19 medication and physical discomfort may also promote psychiatric problems among COVID-19 inpatients (28). This high prevalence of anxiety and depressive symptoms among Bangladeshi COVID-19 inpatients could be because of an inadequate health care system (8), shortage of beds, ICUs, and ventilators (16), treatment-related negligence in health care facilities (29), and less social interaction along with rampant circulation of misinformation on social and conventional media (30).

In this study, the result showed that 78.2% of respondents had physical symptoms after recovery from COVID-19 and weakness (56.6%) was the most prevalent feature. However, a prospective cohort study among the Bangladeshi population reported that the incidence of post-COVID-19 syndrome was 46%, and other features included persistent cough (8.5%), postexertional dyspnea (7%), headache (3.4%), vertigo (2.3%), and sleep-related disorders (5.9%), rates which were lower than those in the present study (31). COVID-19 can cause acute respiratory syndrome with consequent release of proinflammatory cytokines, including interleukin-1β (IL-1β) and IL-6, from the respiratory tract. These cytokines were commonly found to be increased in major depressive disorder (32).

Due to the COVID-19 epidemic, we have had to make some adjustments to our regular schedule. Because of the substantial changes in everyday lives, in this study, 25.2% had suffered from interruption of regular personal life, which has led to concerns in other studies on mental health (3, 16, 23).

The Pearson chi-square test was used in this study to evaluate the association between depression and sociodemographic characteristics with factors related to COVID-19-recovered patients. According to the results, education qualification (*P* = 0.001), diabetes mellitus comorbidity (*P* = 0.001), receiving an antidepressant drug (*P* = 0.006), having fear of further contamination (*P* = 0.003), feeling of being harmful to family members (*P* = 0.001), fear of losing one’s job (*P* = 0.013), being affected by social media posts about COVID-19 (*P* = 0.001), mental distress due to social distancing (*P* = 0.001), and duration of hospitalization (*P* = 0.007) were statistically significantly related to depression (*P* = 0.001), a finding which is consistent with other researches (12, 13, 16). The current study highlights the necessity for reducing this psychological suffering in Bangladesh. Appropriate supportive programs and interventional approaches should be implemented in Bangladesh during the COVID-19 pandemic.

### Limitations.

This study has a few limitations. First, for convenience data were collected from the patient during the COVID-19 recovery period, which could result in recall and selection bias. Second, the risk factors for depression were not able to be analyzed in association with the level of depression. Third, all the respondents in this study were sampled from two selected post-COVID units in Dhaka city, so the generalizability of this study was indeterminate and might not present the whole country’s situation during the COVID-19 pandemic.

### Conclusion.

That the state of depression impacts COVID-19-recovered patients is undeniable. The study found that a significant portion of respondents reported mental health problems, with different levels of severity of depression. Most of the respondents were concerned about the presence of COVID-19-related symptoms, and fear of losing jobs, fear of reinfection, distress due to social distancing, etc., provoked depression in individuals. The findings of the study suggest the need for more targeted measures like health education intervention for people infected with COVID-19 in Bangladesh to accelerate progress in reducing the incidence of depression.

## MATERIALS AND METHODS

### Study design.

This cross-sectional study was conducted among COVID-19-recovered patients, who came for follow-up from 14 days up to 3 months after infection at the post-COVID unit of Dhaka Medical College Hospital (DMCH) and Dhaka North City Corporation Hospital (DNCCH), Dhaka, Bangladesh, from 1 January to 31 December 2021. Patients with known cases of depressive illness and other psychiatric disorders were excluded from this study.

### Sampling and sample size.

A nonprobability convenience sampling method was used, and to estimate the required sample size, the prevalence for depression was set as 47.2% (16). So, the calculated sample size was 382, but because of the unfavorable COVID-19 scenario, it was possible to conduct interviews with only 325 respondents during the designated data collection period.

### Ethical considerations.

Ethical approval for the study was granted by the Institutional Review Board (IRB) of National Institute of Preventive and Social Medicine (NIPSOM) with memo no. NIPSOM/IRB/2021/18 dated 13 December 2021. Written informed consent for participation was required for this study in accordance with national legislation and institutional requirements.

### Data collection.

According to the specific objectives and after the pretest observation, a semistructured questionnaire was developed in English and then translated into Bengali using the selected variables. The respondents were advised of the title and purpose of the study, their anonymity, and voluntary participation in the study by ensuring privacy and confidentiality.

### Data collection instrument.

After informed written consent, the respondents were interviewed face to face with the questionnaire examining sociodemographic variables and the factors causing depression among them. The questionnaire was developed by using the patient health questionnaire (PHQ-9).

### Level of depression.

PHQ-9 has nine different questions that assess the depressive symptoms of the respondents. The total score ranges from 0 to 27 points, where each question is scored from 0 to 3 depending on the answer: 0 (not at all), 1 (several days), 2 (half of the days), and 3 (nearly every day). To determine the state of depression, the total score was divided into four categories: cumulative scores of <10 indicate no depression, 10 to 15 indicate mild depression, 16 to 21 indicate moderate depression, and 22 to 27 indicate severe depression (17).

### Statistical analysis.

Data processing and analysis were done using Statistical Package for Social Sciences (SPSS) version 23 according to objectives and variables. Frequency, percentage, mean, and standard deviation (SD) were used for descriptive statistics, chi-square (χ^2^) tests were carried out to assess the association of qualitative data with 95% confidence interval (CI), and a *P* value of <0.05 was considered statistically significant. Data are presented through tables and figures.

### Data availability.

The original contributions presented in the study are included in the article; further inquiries can be directed to the corresponding author.

## References

[1] World Health Organization (WHO). 2020. Coronavirus. https://www.who.int/health-topics/coronavirus#tab=tab_3. Accessed 15 November 2020.

[2] Director General of Health Services (DGHS). 2020. Corona virus information. Ministry of Health and Family Welfare, Dhaka, Bangladesh. https://corona.gov.bd/. Accessed 13 November 2020.

[3] Liu S, Yang L, Zhang C, Xiang Y-T, Liu Z, Hu S, Zhang B. 2020. Online mental health services in China during the COVID-19 outbreak. Lancet Psychiatry 7:e17–e18. doi:10.1016/S2215-0366(20)30077-8. 32085841 PMC7129099

[4] Kim HC, Yoo SY, Lee BH, Lee SH, Shin HS. 2018. Psychiatric findings in suspected and confirmed Middle East respiratory syndrome patients quarantined in hospital: a retrospective chart analysis. Psychiatry Investig 15:355–360. doi:10.30773/pi.2017.10.25.1. PMC5912494 29593206

[5] Lam MH-B, Wing Y-K, Yu MW-M, Leung C-M, Ma RCW, Kong APS, So WY, Fong SY-Y, Lam S-P. 2009. Mental morbidities and chronic fatigue in severe acute respiratory syndrome survivors: long-term follow-up. Arch Intern Med 169:2142–2147. doi:10.1001/archinternmed.2009.384. 20008700

[6] Rajkumar RP. 2020. COVID-19 and mental health: a review of the existing literature. Asian J Psychiatr 52:102066. doi:10.1016/j.ajp.2020.102066. 32302935 PMC7151415

[7] Sifat RI. 2020. Impact of the COVID-19 pandemic on domestic violence in Bangladesh. Asian J Psychiatr 53:102393. doi:10.1016/j.ajp.2020.102393. 32916443 PMC7462560

[8] Anwar S, Nasrullah M, Hosen MJ. 2020. COVID-19 and Bangladesh: challenges and how to address them. Front Public Health 8:154. doi:10.3389/fpubh.2020.00154. 32426318 PMC7203732

[9] Li W, Yang Y, Liu Z-H, Zhao Y-J, Zhang Q, Zhang L, Cheung T, Xiang Y-T. 2020. Progression of mental health services during the COVID-19 outbreak in China. Int J Biol Sci 16:1732–1738. doi:10.7150/ijbs.45120. 32226291 PMC7098037

[10] Pakpour AH, Griffiths MD. 2020. The fear of COVID-19 and its role in preventive behaviors. J Concurr Disord 2:58–63. http://irep.ntu.ac.uk/id/eprint/39561.

[11] Mak IW, Chu CM, Pan PC, Yiu MG, Chan VL. 2009. Long-term psychiatric morbidities among SARS survivors. Gen Hosp Psychiatry 31:318–326. doi:10.1016/j.genhosppsych.2009.03.001. 19555791 PMC7112501

[12] Deng J, Zhou F, Hou W, Silver Z, Wong CY, Chang O, Huang E, Zuo QK. 2021. The prevalence of depression, anxiety, and sleep disturbances in COVID-19 patients: a meta-analysis. Ann N Y Acad Sci 1486:90–111. doi:10.1111/nyas.14506. 33009668 PMC7675607

[13] Renaud-Charest O, Lui LMW, Eskander S, Ceban F, Ho R, Di Vincenzo JD, Rosenblat JD, Lee Y, Subramaniapillai M, McIntyre RS. 2021. Onset and frequency of depression in post-COVID-19 syndrome: a systematic review. J Psychiatr Res 144:129–137. doi:10.1016/j.jpsychires.2021.09.054. 34619491 PMC8482840

[14] Mamun MA, Hossain MS, Griffiths MD. 2022. Mental health problems and associated predictors among Bangladeshi students. Int J Ment Health Addict 20:657–671. doi:10.1007/s11469-019-00144-8.

[15] Ho CS, Chee CY, Ho RC. 2020. Mental health strategies to combat the psychological impact of coronavirus disease 2019 (COVID-19) beyond paranoia and panic. Ann Acad Med Singap 49:155–160. doi:10.47102/annals-acadmedsg.202043. 32200399

[16] Zubayer AA, Rahman ME, Islam MB, Babu SZD, Rahman QM, Bhuiyan MRAM, Khan MKA, Chowdhury MAU, Hossain L, Habib RB. 2020. Psychological states of Bangladeshi people four months after the COVID-19 pandemic: an online survey. Heliyon 6:e05057. doi:10.1016/j.heliyon.2020.e05057. 33015396 PMC7521899

[17] Das R, Hasan MR, Daria S, Islam MR. 2021. Impact of COVID-19 pandemic on mental health among general Bangladeshi population: a cross-sectional study. BMJ Open 11:e045727. doi:10.1136/bmjopen-2020-045727. PMC8042595 33837107

[18] Xiong J, Lipsitz O, Nasri F, Lui LMW, Gill H, Phan L, Chen-Li D, Iacobucci M, Ho R, Majeed A, McIntyre RS. 2020. Impact of COVID-19 pandemic on mental health in the general population: a systematic review. J Affect Disord 277:55–64. doi:10.1016/j.jad.2020.08.001. 32799105 PMC7413844

[19] Dai L-L, Wang X, Jiang T-C, Li P-F, Wang Y, Wu S-J, Jia L-Q, Liu M, An L, Cheng Z. 2020. Anxiety and depressive symptoms among COVID-19 patients in Jianghan Fangcang Shelter Hospital in Wuhan, China. PLoS One 15:e0238416. doi:10.1371/journal.pone.0238416. 32857826 PMC7454940

[20] Ueda M, Stickley A, Sueki H, Matsubayashi T. 2020. Mental health status of the general population in Japan during the COVID-19 pandemic. Psychiatry Clin Neurosci 74:505–506. doi:10.1111/pcn.13105. 32609413 PMC7361838

[21] Newby JM, O’Moore K, Tang S, Christensen H, Faasse K. 2020. Acute mental health responses during the COVID-19 pandemic in Australia. PLoS One 15:e0236562. doi:10.1371/journal.pone.0236562. 32722711 PMC7386645

[22] Vahedian-Azimi A, Moayed MS, Rahimibashar F, Shojaei S, Ashtari S, Pourhoseingholi MA. 2020. Comparison of the severity of psychological distress among four groups of an Iranian population regarding COVID-19 pandemic. BMC Psychiatry 20:402. doi:10.1186/s12888-020-02804-9. 32770975 PMC7414274

[23] Gao J, Zheng P, Jia Y, Chen H, Mao Y, Chen S, Wang Y, Fu H, Dai J. 2020. Mental health problems and social media exposure during COVID-19 outbreak. PLoS One 15:e0231924. doi:10.1371/journal.pone.0231924. 32298385 PMC7162477

[24] Huang Y, Zhao N. 2020. Generalized anxiety disorder, depressive symptoms and sleep quality during COVID-19 outbreak in China: a web-based cross-sectional survey. Psychiatry Res 288:112954. doi:10.1016/j.psychres.2020.112954. 32325383 PMC7152913

[25] Moghanibashi-Mansourieh A. 2020. Assessing the anxiety level of Iranian general population during COVID-19 outbreak. Asian J Psychiatr 51:102076. doi:10.1016/j.ajp.2020.102076. 32334409 PMC7165107

[26] Wang C, Pan R, Wan X, Tan Y, Xu L, McIntyre RS, Choo FN, Tran B, Ho R, Sharma VK, Ho C. 2020. A longitudinal study on the mental health of general population during the COVID-19 epidemic in China. Brain Behav Immun 87:40–48. doi:10.1016/j.bbi.2020.04.028. 32298802 PMC7153528

[27] Zolotov Y, Reznik A, Bender S, Isralowitz R. 2022. COVID-19 fear, mental health, and substance use among Israeli university students. Int J Ment Health Addict 20:230–236. doi:10.1007/s11469-020-00351-8. 32837432 PMC7299139

[28] Islam MS, Sujan MSH, Tasnim R, Sikder MT, Potenza MN, van Os J. 2020. Psychological responses during the COVID-19 outbreak among university students in Bangladesh. PLoS One 15:e0245083. doi:10.1371/journal.pone.0245083. 33382862 PMC7775049

[29] Al-Zaman MS. 2020. Healthcare crisis in Bangladesh during the COVID-19 pandemic. Am J Trop Med Hyg 103:1357–1359. doi:10.4269/ajtmh.20-0826. 32828138 PMC7543838

[30] Zarocostas J. 2020. How to fight an infodemic. Lancet 395:676. doi:10.1016/S0140-6736(20)30461-X. 32113495 PMC7133615

[31] Mahmud S, Mohsin M, Khan IA, Mian AU, Zaman MA. 2021. Knowledge, beliefs, attitudes and perceived risk about COVID-19 vaccine and determinants of COVID-19 vaccine acceptance in Bangladesh. PLoS One 16:e0257096. doi:10.1371/journal.pone.0257096. 34499673 PMC8428569

[32] Conti P, Ronconi G, Caraffa A, Gallenga C, Ross R, Frydas I, Kritas S. 2020. Induction of pro-inflammatory cytokines (IL-1 and IL-6) and lung inflammation by coronavirus-19 (COVID-19 or SARS-CoV-2): anti-inflammatory strategies. J Biol Regul Homeost Agents 34:327–331. doi:10.23812/CONTI-E. 32171193

